# Influenza A virus shedding and reinfection during the post-weaning period in swine: longitudinal study of two nurseries

**DOI:** 10.3389/fvets.2024.1482225

**Published:** 2024-11-13

**Authors:** Suzanna M. Storms, Antonio Leonardi-Cattolica, Tara Prezioso, Csaba Varga, Leyi Wang, James Lowe

**Affiliations:** ^1^Department of Veterinary Clinical Medicine, University of Illinois at Urbana-Champaign, Champaign, IL, United States; ^2^Department of Pathobiology, University of Illinois at Urbana-Champaign, Champaign, IL, United States

**Keywords:** Influenza A virus, swine, reinfection, disease outbreaks, whole genome sequencing

## Abstract

**Introduction:**

Influenza A virus in swine (IAV-S) is common in the United States commercial swine population and has the potential for zoonotic transmission.

**Objective:**

To elucidate influenza shedding the domestic pig population, we evaluated two commercial swine farms in Illinois, United States, for 7 weeks. Farm 1 had a recent IAV-S outbreak. Farm 2 has had IAV-S circulating for several years.

**Methods:**

Forty post-weaning pigs on Farm 1 and 51 pigs from Farm 2 were individually monitored and sampled by nasal swabs for 7 weeks.

**Results:**

RT-PCR results over time showed most piglets shed in the first 2 weeks post weaning, with 91.2% shedding in week one, and 36.3% in week two. No difference in the number of pigs shedding was found between the two nurseries. Reinfection events did differ between the farms, with 30% of piglets on Farm 1 becoming reinfected, compared to 7.8% on Farm 2. In addition, whole genome sequencing of nasal swab samples from each farm showed identical viruses circulating between the initial infection and the reinfection periods. Sequencing also allowed for nucleic and amino acid mutation analysis in the circulating viruses, as well the identification of a potential reverse zoonosis event. We saw antigenic site mutations arising in some pigs and MxA resistance genes in almost all samples.

**Conclusion:**

This study provided information on IAV-S circulation in nurseries to aid producers and veterinarians to screen appropriately for IAV-S, determine the duration of IAV-S shedding, and predict the occurrence of reinfection in the nursery period.

## 1 Introduction

Influenza A virus in swine (IAV-S) is a common pathogen in US swine herds, and zoonotic and reverse zoonotic transmission makes control efforts challenging (1–4). IAV-S causes systemic signs, such as fever, lethargy, and anorexia, as well as respiratory signs, such as nasal discharge, cough, tracheitis, bronchitis, bronchiolitis, bronchopneumonia, and interstitial pneumonia (5, 6). Most animals recover from the acute viral infection after an illness of 7 days. However, the weekly farrowing of new piglets on commercial sow farms in the US provides a continuous naive population for IAV to infect, in addition to unvaccinated/unexposed replacement gilts (7). Direct contact between infected and susceptible animals is thought to be the most likely method of infection. However, transmission by indirect contact and aerosols are also documented, and persistent viral presence in swine systems is attributed to housing, intrafarm movement of animals, and stocking density (8, 9).

Several studies in recent years focused on IAV detection and prevention in farrowing, as well as the peri- weaning period (10–12). Their findings show that maternally derived antibodies (MDA) are important for protecting neonates from pathogens early in life, but MDA can interfere with vaccine efficacy, resulting in incomplete protection and increased IAV shedding (13, 14). Colostrum is necessary for optimal piglet health, due to the naïve state of the immune system at birth (15). In the United States, commercial pigs are often weaned at 3 weeks of age and raised in multi-stage production systems, which include transportation to a nursery facility or a wean-to-finish site. There, pigs are mixed in pens that hold 25–200 head, introducing a variety of pathogens to the vulnerable population of weaned pigs. Weaned pigs remain in the nursery until about 10 weeks of age, and during this time, MDA wane and piglet-derived antibodies increase as their immune system is challenged (16).

The diversity of IAV in swine (IAV-S), driven by antigenic drift and shift, presents the greatest challenge to producing vaccines that effectively mitigate infection and disease. Brooke reviewed IAV diversity and suggested that each newly synthesized IAV viral genome (~13 kb) could contain an average of up to two mutations (17). Such genomic instability creates challenges when producing commercial vaccines and may account for the large number of autogenous and prescription vaccine platforms available for swine producers. Despite the “moving target” of genomic variation, vaccination remains the primary medical prevention method available for IAV-S (18). However, in the 2021 NAHMS Swine Large Enterprise Survey (farms greater than 1,000 head), 54% of producers vaccinated sows for IAV-S, while 21.6% vaccinated growing pigs (19). This provides a dearth of data in which to interpret preventive methods, and more research is needed to assess which vaccination strategies are the most efficacious in preventing and limiting disease in IAV-S.

In this study, we observed piglets from two naturally infected commercial swine farms in the midwestern United States. The study’s first objective was to determine when weaned piglets shed IAV, and to what extent they became reinfected during the post-weaning period. The second objective was to observe the viral diversity during this period. We serially sampled piglets weekly for 7 weeks in two separate nurseries and measured IAV shedding from nasal swabs. We also performed direct whole genome sequencing (WGS) on positive nasal swab samples to observe viral diversity and change during the nursery phase of production. We noted highest shedding rates in the first week post-weaning, with additional shedding events occurring in weeks 6 and 7. This shedding information can help direct surveillance efforts by targeting specific ages, thus enhancing efficiency, and decreasing labor and testing expenses. Our WGS analysis showed that Farm 1 was infected with the H1N1 1A.3.3.2 pandemic clade, while Farm 2 fell into the 1A.3.3.3c-3 gamma-c3 clade. WGS analysis showed independent nucleotide changes in antigenic sites as well as conserved mutations that were previously reported to confer myxovirus resistance gene A (MxA) resistance. Our study suggests early sampling times for detecting IAV-S in weaned pigs, describes infection and reinfection events, and identified the genetic diversity of IAV within farms.

## 2 Methods

### 2.1 Setting, study design, and definitions

This study was performed longitudinally, observing two groups of 60 piglets on two separate farms in the state of Illinois, United States. The sampling period was from January to May 2022. Both farms in this study were sampled after the piglets were co-mingled within the first 2 days of weaning.

#### 2.1.1 Farm 1 description

Farm 1 is a 3,000-head multiplier sow farm with an attached gilt development unit. This farm provided a controlled environment that limited the introduction of other influenza strains and other infectious diseases. This farm was chosen for this study because the researchers were interested in the infection dynamics of IAV-S in a previously uninfected herd. This sow farm has 6–8 full-time employees that care for the animals daily. This farm had an outbreak of a pandemic H1N1 strain in October of 2021 but was influenza-free for over 5 years before the introduction. The IAV-S outbreak was discovered in the sows during early gestation of the piglets sampled in the study. We assume that the sows naturally exposed to virus during early gestation produced virus-specific antibodies to the circulating virus. Maternally derived antibodies would then pass to their litters via colostrum at parturition, although piglet antibody data will be presented in a different analysis.

It is unknown how the strain entered the farm. The farm is relatively isolated, and no new animals have been introduced (all replacement gilts are produced on-site). This study utilized the replacement gilts chosen by the farm as replacement females based upon a genetic selection index and several phenotypic traits and housed in a gilt development unit, with rooms holding approximately 75 head at placement. Gilts were chosen the day after weaning (3 weeks of age) from one room, and the first samples were collected. The farm was PRRS negative during the sampling period.

##### 2.1.1.1 Farm 1 vaccination

Sow Farm 1 uses the following commercial vaccines during each gestation or post farrow: FluSure XP® (Zoetis, Kalamazoo, MI, United States), Prosystem RCE (Merck Animal Health, Rahway, NJ, USA), FarrowSure® GOLD (Zoetis, Kalamazoo, MI, USA). The sows receive FluSure XP® during week 5 of each gestation, and replacement gilts receive two doses of FluSure XP® at 20 and 24 weeks post-weaning in the GDU. Replacement gilts also receive the following commercial vaccines in the GDU: Circumvent® PCV-M G2, Porcilis® Ileitis (Merck Animal Health, Rahway, NJ, United States) (during the study period), FarrowSure® Gold (after study period).

The FluSure XP® vaccine (Zoetis, Kalamazoo, MI, United States) includes hemagglutinin antigens for H3 clusters IV-A and IV-B, as well as H1N2 (delta) and an H1N1 (gamma), when screened by the ISUFluture HA identity tool and NCBI nucleotide blast search (20, 21).

#### 2.1.2 Farm 2 description

Farm 2 is a commercial nursery facility where weaned piglets are transported (approximately 2 h) from the sow farm of origin. This nursery is a 4,000 space facility; four barns with 1,000 spaces in each barn. Here, the piglets remain for 10 weeks post-weaning. Each barn is filled and emptied in an all-in, all-out fashion. Importantly though, different barns at the facility are turned over at different times. One person manages the day-to-day care of all animals in the nursery. Farm 2 piglets were weaned (3 weeks old) mixed-sex commercial gilts and barrows, and four piglets were tagged per sow by farm workers before being transported to the nursery facility. Piglets were sampled the day after placement. Around 150 piglets were housed in each nursery pen and shared the airspace within the barn. Piglets in the study were occupants of two pens.

##### 2.1.2.1 Farm 2 vaccination

Sow Farm 2 used autogenous flu vaccine. The IAV-S vaccine was a prime-boost killed vaccine consisting of two quadrivalent killed vaccines for eight strains (four in primary vaccine for gilts, four others in booster vaccine in sows). Replacement gilts are vaccinated with the autogenous vaccine at 19 and 21 weeks post weaning, and sows are each boosted during mid-lactation. The vaccine included isolates obtained from the swine system, of subtypes H1N1, H1N2, and H3N2, containing HA clusters of H1 (alpha, gamma, pandemic (pdm), delta 1, delta 1B, delta 2), and H3 (1, IVA). Strains included in the vaccine were updated regularly when detected on the farm. Sows of different ages would have received slightly different vaccines depending on the autogenous vaccine being updated annually. However, each would cover each of these clades.

The sow farm also uses the following commercial vaccines during each gestation during lactation: Prosystem RCE (Merck Animal Health, Rahway, NJ, United States), FarrowSure® GOLD (Zoetis, Kalamazoo, MI, USA). Replacement gilts received the following commercial vaccines prior to entry on the sow farm: Circumvent® PCV-M G2, Enterisol® Ileitis and Ingelvac® ERY-ALC (Boehringer Ingelheim, Duluth, GA, United States) FarrowSure® Gold.

Piglets were not vaccinated for IAV, and only received natural passive immunity from their dams via colostrum.

### 2.2 Infectious disease case definition, ethical statement, participants, sample collection

All sampling methods and protocols complied with the University of Illinois Institutional Animal Care and Use Committee (No. 19199) and the Institutional Biosafety Committee guidelines. After collection, all samples were processed in a Biosafety Level 2 (BSL-2) laboratory.

The sample size was restricted to the number of animals kept as replacement gilts in the gilt development unit (GDU) on Farm 1. The farm selects approximately 75 females to enter the GDU each weaning period. We estimated that 75% of the animals would be infected and selected a desired precision of 0.05 and confidence level of 0.9, which estimated 55 animals needed to be included in the study (22). We expected 10% loss to follow-up, so 60 animals were enrolled on each farm.

Sixty animals from each farm were enrolled, and data from 91 of the 120 pigs (40 from Farm 1 and 51 from Farm 2) were complete at all timepoints and included in this study. Each animal was given an ear tag for identification. Pigs were individually nasal swabbed weekly beginning within the first 2 days following weaning. Pigs were restrained manually or using a snare (when they were too large for manual restraint). A single polyester mini-tip swab on an aluminum shaft (Puritan™ 25,800 D 50) was inserted in both nares as caudally as possible (to ethmoid turbinates). Swab tips were cut and inserted into 2 mL cryotubes containing 1.8 mL of BHI (brain-heart infusion) viral transport media with penicillin G and streptomycin sulfate (23). The tubes were placed on ice for transport to the lab (about 3 h), where they were placed at −80°C for long-term storage until processing. A total of 91 piglets with complete data were included in this study amounting to 637 nasal swabs.

In this study, isolate means a virus sampled from the farm for further use in the lab or for vaccine production. Shedding indicates a nucleic acid positive nasal swab as detected by real time reverse transcription polymerase chain reaction (RT-PCR). We assume that each pig is or was infected if RT-PCR detected IAV genetic material. However, we make no assertion they were diseased, as clinical signs were not assessed. Reinfection refers to an animal that had at least two shedding events, with at least 1 week of no genomic material detected between shedding events.

### 2.3 Laboratory methods

#### 2.3.1 RNA extraction

All samples were processed in a BSL-2 lab, and all potentially infectious samples were processed in a biosafety cabinet. The nasal swabs were moved to the biosafety cabinet, thawed, and vortexed for 1 min. 200 μL of sample was used for each reaction. The MagMAX™ Viral/Pathogen Nucleic Acid Isolation kit was used for extraction using a Kingfisher Flex instrument (ThermoFisher Scientific, Waltham, MA, USA). We used the low sample volume modification according to the manufacturer’s protocol, which uses half as much binding solution, wash solution, elution solution, proteinase K, and DNA/RNA binding beads, and is used for 200 μL of sample. Complete extraction details can be found in section 1.1 of the Supplementary materials.

#### 2.3.2 RT-PCR of nasal swab samples

The USDA licensed VetMAX™-Gold SIV Detection kit (Applied Biosystems, Waltham, MA United States) was used for influenza genome detection in extracted samples per manufacturer’s instructions. Complete RT-PCR methods can be found in the Supplementary materials section 1.2. All PCR preparation was performed in a dedicated area with dedicated pipettes to reduce the incidence of contamination.

After the PCR amplification, data analysis was performed to normalize the data per the manufacturer’s instructions. The raw data files were exported, and the ΔRn values of the two positive control samples at cycle 40 were averaged (maximum fluorescence values) for the FAM and VIC channels. In the QuantStudio Design and Analysis software, a manual threshold for each channel was set to be 5% of the average maximum fluorescent value of the positive control amplification signals. The data was then re-analyzed using the manually adjusted threshold value, and the normalized PCR Cq values were used for analysis. Per the manufacturer’s instructions, samples with a Cq value of 38.0 and above were considered negative. Samples with RT-PCR Cq values less than 25 were submitted for WGS. During sequencing analysis, we saw only one strain circulating on each farm.

#### 2.3.3 Whole genome sequencing

WGS was performed on fresh RNA extracts as described above. An enrichment step targeting each segment of the influenza genome was performed with modified primers previously published and can be found in Supplementary Table S1 (24–26). The SuperScript™ III One-Step RT-PCR System with Platinum™ Taq High Fidelity DNA Polymerase (Invitrogen, Waltham, MA, United States) was used according to the manufacturer’s instructions. Briefly, 25 μL of the 2x reaction mix, 1uL of the primer mix, 1uL of the Superscript III RT/Taq enzyme, and 3 μL of RNase/DNase free water were used in each reaction and loaded into MicroAmp (Applied Biosystems, Waltham, MA, United States) tube strips. 20 μL of freshly extracted RNA was added to each reaction tube.

The thermocycler program was as follows: Reverse transcription at 42°C for 1 h, followed by RT inactivation/initial denaturation at 94°C for 2 minutes. The first PCR cycle, amplifying the HA, NP, NA, and M segments, was as follows: 94°C for 30 s, 45°C for 30 s, 68°C for 7 min, for 20 cycles. The second PCR step, amplifying the PB2, PB1, PA, and NS genes, was as follows: 94°C for 30 s, 57°C for 30 s, and 68°C for 7 min for 20 cycles. The final elongation was at 68°C for 5 min, followed by a hold step at 4°C until the tubes were removed from the thermocycler. DNA purification was performed using the QIAquick PCR purification (Qiagen, Venlo, Netherlands) according to the instructions in the Supplementary materials S1.3.

A Qubit™ dsDNA Quantification Assay kit (ThermoFisher Scientific, Waltham, MA, United States) was used to quantify cDNA and dilute samples to the correct concentration for the library preparation. The library preparation was performed using the Nextera XT DNA Library Preparation Kit per Illumina’s Nextera XT DNA library prep reference guide. A total amount of 5 ng of purified PCR product was used for the next-generation sequencing. Library preparation and NGS on the Illumina MiSeq platform were conducted by the University of Illinois Veterinary Diagnostic Laboratory.

#### 2.3.4 Consensus sequence assembly

FASTQ data obtained from the MiSeq instrument were processed on the EU Galaxy server (usegalaxy.eu), following the Avian influenza viral strain analysis from the gene segment sequencing data training material (27–29). Paired end FASTQ files from each sample were processed by fastp for initial pre-processing with sequences lengths <30 discarded (30). Reads with a mean quality below 30 were cut from the 5′ and the 3′ reads. Next, the processed FASTQ files were run with VAPOR, a tool for influenza classification for short read data, which identifies references to be used for mapping and assembly from a customizable database of 50 references (31). The Kmer filtering threshold was set to 0.1, and the minimum Kmer proportion was set to 0.0. The top scoring matches for each segment were used for mapping using BWA-MEM for each gene segment, using default settings (32). Samtools view was used to filter mapped reads to keep only those with quality scores above 20, and were paired reads with proper pairs (33). Mapping statistics were generated using QualiMap BamQC (34, 35). Next the BAM files were split by the references selected earlier, using BamTools (36). The consensus sequence was generated per segment using the iVar consensus tool (37). The minimum quality score threshold to count base was set at 20, the minimum frequency threshold at 0.7, the minimum indel frequency threshold at 0.8, and the minimum depth to call consensus was set at 10 for the first pass. For sequences that had ambiguous bases, the frequency threshold was decreased to 0.5, with a minimum depth to call consensus set at 2. The complete workflow can be found at: https://training.galaxyproject.org/training-material/topics/variant-analysis/tutorials/aiv-analysis/tutorial.html. Assembled gene segments of each strain were deposited into GenBank and their accessions can be found in the Supplementary Table S2.

#### 2.3.5 Sequence analysis

Following consensus sequence generation, the sequences of each segment were aligned using MAFFT (38, 39). Aligned sequences were translated into amino acids in MEGA (version 11) (40), and non-synonymous mutations were recorded by location, change, and frequency.

##### 2.3.5.1 Phylogenetic tree generation

Fifty nine reference sequences of representative HAs were chosen from the USDA APHIS Swine Surveillance quarterly reports from 2021 to 2023, as well as one classic strain to root the tree (41). The HA reference sequences were chosen by PARNAS, a tool built by the National Animal Disease Center in Ames, IA, United States, which selects the most representative taxa and downsamples large phylogeny while preserving diversity, reducing redundancy among sequences, and identifying key diversity groups in a phylogeny (42). The HA sequences from Farms 1 and 2 were aligned with the 59 references using MAFFT, and a maximum likelihood tree was chosen using IQ-TREE and bootstrapped 1,000 times (43–46). Trees were visualized using FigTree (version 1.4,4) and the reference sequences tree branches were then classified by the OctoFluShow database, which has pre-determined clade and constellation designations for over 11,000 IAV-S strains, and annotated in FigTree (47).

Similarly, sequences were downloaded from BV-BRC.org to generate a reference phylogenetic tree to classify the other seven gene segments from the farms. BV-BRC sequences were filtered by completeness, isolation country (United States), host common name (pig), and collection year (2013–2024), and sequences were downloaded by segment on April 11, 2024.

PARNAS was used to select representative sequences for NA and the internal genes using a similarity threshold of 96% (42). Again, PARNAS-selected representative sequences for the other seven gene segments were aligned and treed, and the strains used in the final trees can be found in Supplementary Table S3. The PARNAS-selected and Farm sequences were then aligned using MAFFT, and a maximum likelinood tree whas chosen, as stated above. The OctoFluShow database was used to classify and annotate the Farms’ HA and NA clades and internal gene constellations according to where the segments treed in relation to the assigned clades of the PARNAS-selected reference strains.

### 2.4 Statistical analysis

The shedding data was explored and cleaned. Reinfected piglets were identified based on a positive shedding variable. Any piglet that was positive, then negative, for any one or more week-periods, followed by another positive RT-PCR was considered reinfected.

Two mixed-effects logistic regression models were constructed using Generalized Linear Mixed Models with adaptive Gaussian quadrature, and including the pigs as random intercepts. For these analyses, the GLMMAdaptive package (81) in R Studio (80) using the R language (79) was used. The first model included reinfection (Yes = 1; No = 0) as the binary outcome variable, and the second model included shedding (Yes = 1; No = 0) as the binary outcome variable.

The first model included 3 predictor variables. A categorical variable representing the week of sampling (weeks 1–7), including the first week as the reference to which all the other weeks were compared. The second and third predictor variables represented the status of virus shedding (Yes = 1; No = 0), and farms (Farm1 vs. Farm2), respectively. In the first step, univariable models were constructed, and only variables significant at *p* ≤ 0.05 were included in the multivariable model. The final mixed-effect multivariable model only included the outcome variable and the significant predictors (week and shedding), as the fixed effects, and the pigs as random intercepts to account for clustering.

The following formula defines the model:


$$\begin{array}{l} \mathrm{logit}\left(P\left(\mathrm{Reinfectio}n_{i}|\mathrm{Wee}k_{ij},\mathrm{She}d_{ij},b_{i}\right)\right)=\\ \beta 0+\beta 1\;\mathrm{week}2+\beta 2\;\mathrm{week}3+\beta 3\;\mathrm{week}4+\\ \beta 4\;\mathrm{week}5+\beta 5\;\mathrm{week}6+\beta 6\;\mathrm{week}7+\beta 7\;\mathrm{shed}+b_{i} \end{array}$$

Where:


$$b_{i}\sim N\left(0,1\right)$$

The second model included 2 predictor variables: the week of sampling (weeks 1–7), and the farm. During univariate analysis, the farm predictor variable was not significant. Therefore, the final model only included the shedding outcome variable and the week of sampling predictor, and pigs as random intercepts.

The following formula defines the model:


$$\begin{array}{l} \mathrm{logit}\left(P\left(\mathrm{Shed}|\mathrm{Wee}k_{ij},b_{i}\right)\right)=\beta 0+\beta 1\;\mathrm{week}2+\beta 2\;\mathrm{week}3+\\ \beta 3\;\mathrm{week}4+\beta 4\;\mathrm{week}5+\beta 5\;\mathrm{week}6+\beta 6\;\mathrm{week}7+b_{i} \end{array}$$

Where:


$$b_{i}\sim N\left(0,1\right)$$

For both models, odds ratios were calculated by exponentiating the model estimates. An OR > 1 signifies increased probability, while an OR < 1 signifies decreased probability.

The marginal coefficients and their standard errors for the predictor variables were calculated based on a Monte Carlo procedure using the GLMMAdaptive package.

The goodness of fit for both final models was assessed using the DHARMa package (82).

## 3 Results

### 3.1 Participants

**Figure 1:**
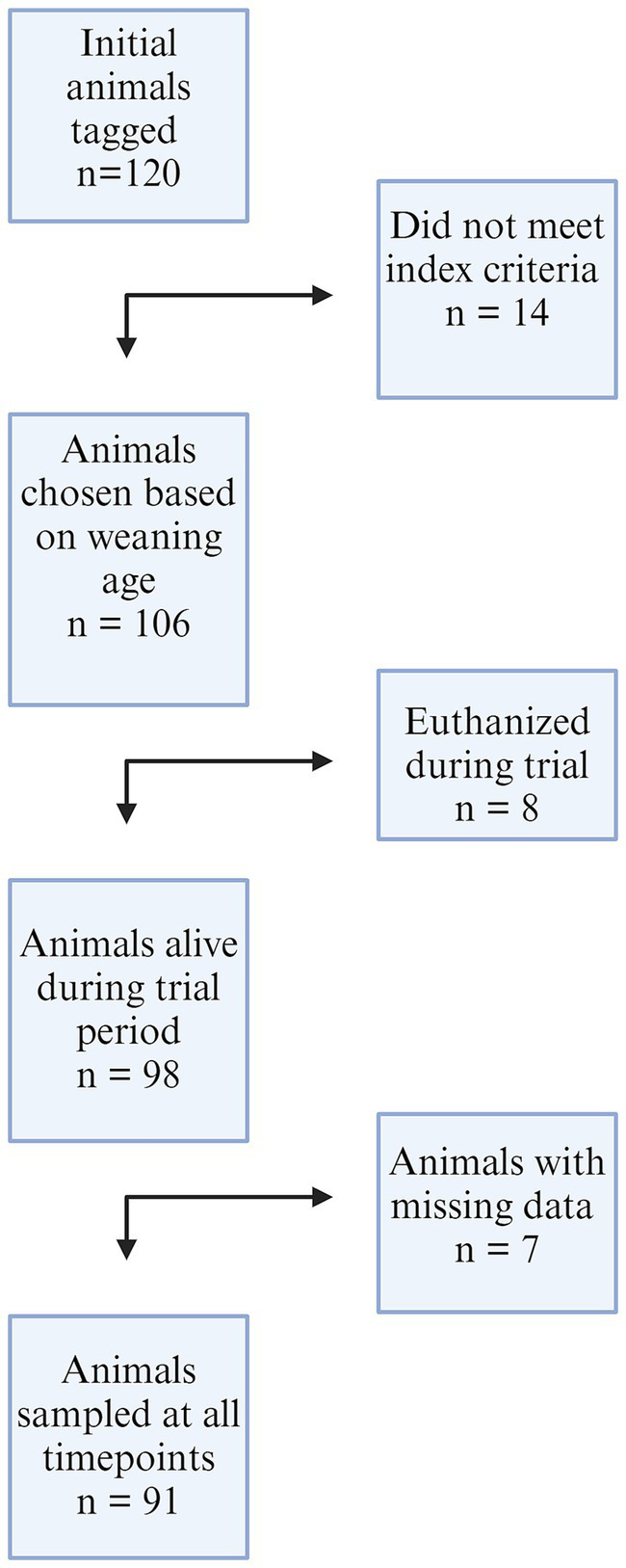
A flowchart of animals included in the study from both farms. One hundred twenty animals were initially enrolled, 14 were removed from Farm 1 due to gilt selection index criteria and weaning age. Eight piglets from both farms were euthanized by farm staff during the trial, and seven animals were removed from the dataset due to incomplete nasal swab results at all timepoints.

One hundred twenty piglets were enrolled during week 1 of the study. Fourteen of these were deemed ineligible due to timing of weaning that did not match the other study participants (pigs 1–14 on Farm 1). Eight pigs were euthanized during the study (26, 42, 49, 60 from Farm 1; 12, 29, 30, 41, 42 from Farm 2) unrelated to study procedures but may have been secondary to respiratory disease. Euthanasia was performed by farm staff when researchers were absent. Six pigs were excluded from the study due to incomplete nasal swab data during all seven sampling periods (19, 57, on Farm 1; 8, 16, 21, 39 on Farm 2). Ninety-one pigs with complete data sets were included in the final analysis. The decision flowchart is seen in Figure 1. All piglet data can be found in Supplementary Table S4.

### 3.2 Prevalence of IAV and RT-PCR analysis

**Figure 2:**
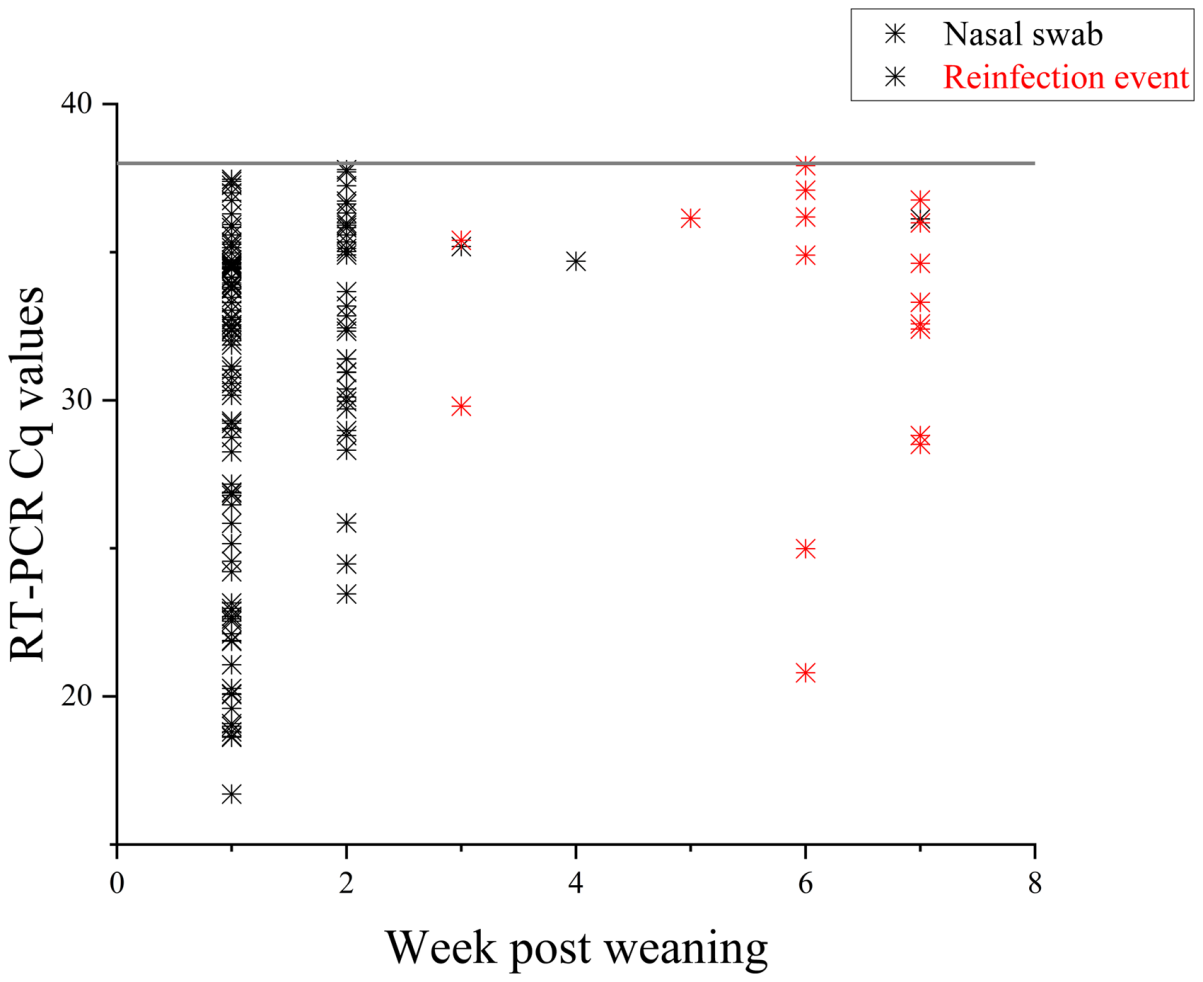
RT-PCR Cq values of nasal swabs taken for 7 weeks post weaning. Positive samples are displayed in the figure. Black stars are initial infections, with durations of one to 3 weeks. Pigs with reinfection events, defined as an initial shedding event followed by at least 1 week with no shedding, are in red. Sixteen pigs had reinfection events.

In total, 637 PCR samples were included in the analysis. One hundred thirty seven samples were positive during the sampling period (21.5%). There was no difference in initial number of pigs shedding events between the two farms (*p* = 0.923). Week 1 had the highest likelihood of shedding (*p* = < 0.001) compared to any other week. Most of the piglets, 97.8% (89/91), shed at least once during the seven-week sampling period. During the 7 weeks, Farm 1 had 95% (38/40) pigs shed and Farm 2 had 100% (51/51) shed. RT-PCR nasal swab detection by week can be found in Tables 1, 2, and Supplementary Table S4. Week 1 had the highest percentage of shedding (83/91), followed by week 2 (33/91). Weeks 3–5 had few shedding events at both farms. Farm 1 revealed increased shedding during week 6 (6/40), and shedding was detected on both farms in week 7 (9/91). Figure 2 displays the RT-PCR Cq values by week and reinfection events. Descriptive statistics of initial shedding and reinfections by week and farm can be found in Tables 3, 4. Shedding and reinfection tables with Cq values by week can be found in Tables 1, 2.

**Table 1:** Weekly nasal swab results for each pig from Farm 1.

Farm 1	Week 1	Week 2	Week 3	Week 4	Week 5	Week 6	Week 7
Pig ID							
15	30.77	39.01	40.00	40.00	40.00	40.00	40.00
16	35.93	40.00	40.00	40.00	36.15	40.00	33.31
17	40.00	39.77	40.00	40.00	40.00	40.00	36.12
18	32.79	40.00	40.00	40.00	40.00	40.00	40.00
20	35.27	40.00	40.00	40.00	40.00	24.99	40.00
21	31.87	30.11	40.00	40.00	40.00	40.00	28.51
22	34.14	40.00	40.00	40.00	40.00	37.10	40.00
23	34.16	40.00	40.00	40.00	40.00	37.92	40.00
24	25.16	35.91	40.00	38.99	40.00	40.00	40.00
25	34.48	23.46	40.00	40.00	40.00	34.90	40.00
27	33.29	28.81	40.00	40.00	40.00	40.00	32.40
28	35.34	40.00	40.00	40.00	40.00	40.00	32.58
29	35.83	37.24	40.00	40.00	40.00	40.00	40.00
30	16.70	35.98	40.00	40.00	40.00	40.00	40.00
31	37.26	35.12	40.00	40.00	40.00	40.00	39.35
32	34.74	36.63	40.00	40.00	40.00	40.00	40.00
33	37.39	40.00	40.00	40.00	40.00	40.00	34.63
34	33.90	40.00	40.00	40.00	40.00	40.00	40.00
35	33.04	40.00	40.00	40.00	40.00	40.00	40.00
36	35.58	24.48	40.00	40.00	40.00	40.00	40.00
37	34.52	40.00	40.00	40.00	40.00	40.00	40.00
38	34.15	40.00	40.00	40.00	40.00	40.00	40.00
39	38.68	40.00	40.00	40.00	40.00	40.00	40.00
40	32.41	40.00	40.00	40.00	40.00	38.10	40.00
41	34.42	40.00	40.00	40.00	40.00	40.00	40.00
43	19.08	40.00	40.00	40.00	40.00	40.00	40.00
44	34.44	40.00	40.00	40.00	40.00	40.00	40.00
45	20.27	40.00	40.00	40.00	40.00	40.00	40.00
46	33.30	40.00	40.00	40.00	40.00	40.00	40.00
47	33.76	40.00	40.00	40.00	40.00	40.00	40.00
48	35.16	40.00	40.00	40.00	40.00	40.00	28.81
50	39.49	40.00	40.00	34.69	40.00	40.00	40.00
51	40.00	40.00	40.00	40.00	40.00	40.00	40.00
52	28.74	40.00	40.00	40.00	40.00	40.00	40.00
53	38.24	40.00	40.00	40.00	40.00	20.80	40.00
54	36.75	40.00	40.00	40.00	40.00	40.00	40.00
55	32.17	40.00	40.00	40.00	40.00	36.19	40.00
56	30.33	25.86	35.19	40.00	40.00	40.00	40.00
58	37.00	40.00	40.00	40.00	40.00	40.00	40.00
59	34.52	40.00	40.00	40.00	40.00	40.00	40.00

Cq values considered positive (<38.00) are highlighted in red. Cq values of 40.00 were negative at the last cycle (40). Piglets that never shed during the study period hare highlighted in green.

**Table 2:** Weekly nasal swab results for each pig from Farm 2.

Farm 2	Week 1	Week 2	Week 3	Week 4	Week 5	Week 6	Week 7
Pig ID							
1	33.47	40.00	40.00	40.00	40.00	40.00	40.00
2	31.15	40.00	40.00	40.00	39.55	40.00	40.00
3	32.35	40.00	29.80	40.00	40.00	40.00	40.00
4	38.68	28.31	38.35	40.00	40.00	40.00	40.00
5	29.05	40.00	40.00	40.00	40.00	40.00	40.00
6	32.55	40.00	40.00	40.00	40.00	40.00	40.00
7	32.00	40.00	40.00	40.00	40.00	40.00	40.00
9	34.97	35.58	40.00	40.00	40.00	40.00	40.00
10	29.30	40.00	40.00	40.00	40.00	40.00	40.00
11	32.73	36.73	40.00	40.00	40.00	40.00	40.00
13	34.64	35.02	40.00	40.00	40.00	40.00	40.00
14	22.97	40.00	40.00	40.00	40.00	40.00	40.00
15	37.46	32.34	40.00	40.00	40.00	40.00	40.00
17	34.57	40.00	40.00	40.00	40.00	40.00	40.00
18	30.57	40.00	40.00	40.00	40.00	40.00	40.00
19	32.53	30.38	40.00	40.00	40.00	40.00	40.00
20	26.90	37.71	40.00	40.00	40.00	40.00	40.00
22	22.12	36.32	40.00	40.00	40.00	40.00	40.00
23	19.00	40.00	40.00	40.00	40.00	40.00	40.00
24	22.88	40.00	40.00	40.00	40.00	40.00	40.00
25	21.89	40.00	40.00	40.00	40.00	40.00	40.00
26	29.04	30.95	40.00	40.00	40.00	40.00	40.00
27	19.60	40.00	40.00	40.00	40.00	40.00	40.00
28	31.02	32.45	40.00	40.00	40.00	40.00	39.61
31	18.65	34.91	40.00	40.00	40.00	40.00	40.00
32	20.06	29.71	40.00	40.00	40.00	40.00	40.00
33	24.21	30.94	40.00	40.00	40.00	40.00	40.00
34	22.71	40.00	40.00	40.00	40.00	40.00	40.00
35	34.61	40.00	40.00	40.00	40.00	40.00	40.00
36	18.62	40.00	40.00	40.00	40.00	40.00	40.00
37	26.79	29.96	40.00	40.00	40.00	40.00	39.68
38	26.87	31.39	40.00	40.00	40.00	40.00	40.00
40	28.25	40.00	40.00	40.00	40.00	40.00	40.00
43	24.56	40.00	40.00	40.00	40.00	40.00	40.00
44	22.54	33.67	40.00	40.00	40.00	40.00	40.00
45	20.09	40.00	40.00	40.00	40.00	40.00	40.00
46	18.80	40.00	35.40	40.00	40.00	40.00	40.00
47	21.86	35.83	40.00	40.00	40.00	40.00	40.00
48	27.17	40.00	40.00	40.00	40.00	40.00	40.00
49	21.07	35.35	40.00	40.00	40.00	40.00	40.00
50	29.22	40.00	40.00	40.00	40.00	40.00	40.00
51	30.17	32.85	40.00	40.00	40.00	40.00	40.00
52	36.30	40.00	40.00	40.00	40.00	40.00	40.00
53	22.62	40.00	40.00	40.00	40.00	40.00	40.00
54	33.96	28.98	40.00	40.00	40.00	40.00	40.00
55	40.00	31.40	40.00	40.00	40.00	40.00	36.76
56	39.67	37.79	40.00	40.00	40.00	40.00	40.00
57	23.16	40.00	40.00	40.00	40.00	40.00	40.00
58	33.81	40.00	40.00	40.00	40.00	40.00	40.00
59	26.46	33.18	40.00	40.00	40.00	40.00	40.00
60	25.85	40.00	40.00	40.00	40.00	40.00	35.99

Cq values considered positive (<38.00) are highlighted in red. Cq values of 40.00 were negative at the last cycle (40). All pigs shed once during the study period.

**Table 3:** Descriptive statistics between reinfected groups and non-reinfected groups stratified by the predictor variables.

	Stratified by reinfection				
	Level	No reinfection	Reinfection	*p*- value	Test
*n*		621	16		
Week of sampling by reinfection *n* = # of pigs that were not reinfected/reinfected that week. (% out of Total in that strata)	1	91 (14.7)	0 (0.0)	<0.001	Fisher
	2	91 (14.7)	0 (0.0)		
	3	89 (14.3)	2 (12.5)		
	4	91 (14.7)	0 (0.0)		
	5	90 (14.5)	1 (6.2)		
	6	86 (13.8)	5 (31.2)		
	7	83 (13.4)	8 (50.0)		
Samples positive during study (% of samples- same as above)	0 - negative	500 (80.5)	0 (0.0)	<0.001	Fisher
	1 - positive	121 (19.5)	16 (100.0)		
Samples per farm (% of samples- same as above)	1	268 (43.2)	12 (75.0)	0.019	Fisher
	2	353 (56.8)	4 (25.0)		

Number of positive samples are presented, followed by percentage of samples by group in parentheses. Due to the low number of sample size in some groups, the Fisher’s test was used to assess the difference between groups. The *p*- value of the Fisher’s test is included. Most reinfection events occurred in weeks six and seven.

**Table 4:** Descriptive Statistics between shedding groups and non-shedding groups stratified by the predictor variables.

	Stratified by Shedding				
	Level	No shedding	Shedding	*p*- value	Test
n		500	137		
Week of sampling (%) *n* = # of pigs that were not shedding/shedding that week (% out of Total in that strata)	1	8 (1.6)	83 (60.6)	<0.001	Chi^2
	2	58 (11.6)	33 (24.1)		
	3	87 (17.4)	4 (2.9)		
	4	90 (18.0)	1 (0.7)		
	5	90 (18.0)	1 (0.7)		
	6	85 (17.0)	6 (4.4)		
	7	82 (16.4)	9 (6.6)		
Samples positive during study (% of samples- same as above)	0 - negative	500 (100.0)	121 (88.3)	<0.001	Fisher
	1 - positive	0 (0.0)	16 (11.7)		
Samples per farm (% of samples- same as above)	1	219 (43.8)	61 (44.5)	0.923	Fisher
	2	281 (56.2)	76 (55.5)		

Number of positive samples are presented, followed by percentage of samples by group in parentheses. Due to the low number of sample size in some groups, the Fisher’s test was used to assess the difference between groups. The *p*- value of the Fisher’s test is included.

#### 3.2.1 Reinfection events

Sixteen piglets had a reinfection event, defined as two shedding events separated by at least 1 week of PCR negative swabs. Farm 1 had 12 reinfection events occurring in weeks 5, 6 and 7, while Farm 2 had four in weeks 3 and 7. One pig became reinfected twice (Farm 1, pig 16), and one pig shed for 3 weeks consecutively (Farm 1, pig 56). Shedding and reinfection tables with Cq values by week can be found in Tables 1, 2. Pigs from Farm 1 were more likely to become reinfected compared to Farm 2 (*p* = 0.019). Descriptive statistics of shedding and reinfections by week and farm can be found in Tables 3, 4. Shedding and reinfection tables with Cq values by week can be found in Tables 1, 2.

### 3.3 Whole genome sequence results

Ten whole genome sequences from Farm 1, and 22 from Farm 2, were obtained with sufficient completeness and were used in the analysis. Twenty five samples were from week 1, four from week 2, one from week 3, and two from week 6. Each segment was assessed for quality and only the segments that met the more stringent threshold frequency of 0.7 and minimum depth of 10 were used in the analysis, as displayed in Supplementary Table S5.

On Farm 1, the consensus sequences were nearly identical. Amino acid identity across all gene segments was greater than 99.3% for all segments, signifying very few mutations in the quasispecies and no reassortant events, and can be seen in Table 5. Analysis of individual amino acid changes showed that the HA had four amino acid mutations, and NP had three. PB2, PA, and NA each had one mutation. The H3 and N2 numbering systems were used for the analysis in order to be consistent with previous publications numbering, and allows for cross-subtype comparison, as proposed by Burke and Smith (48). The HA Subtype Numbering Conversion tool from BV-BRC.org was used to standardize the HA numbering (49). The N2 numbering system was used based upon the WHO N2 numbering scheme to record amino acid substitutions for neuraminidase inhibitors (50).

**Table 5:** Farm 1 Non-synonymous amino acid mutations.

Segment	Mutations Present	Samples with mutation	% Identity across Segment
1 – PB2	D9N	24, 30, 53, 56	99.9%
3 – PA	V100A	53	99.9%
4 – HA	S146G	24	99.3%
	K149N	25	
	T244K	24	
	M260I	24, 30	
5 – NP	D53E**	24, 30	99.4%
	R98K*	24, 30	
	100I*	ALL	
	313 V*	ALL	
	M440L	24, 30	
6 – NA	S379R	53	99.8%

*Indicates MxA resistance. **Indicates reduced virulence. Numbering is based on the H3 and N2 numbering systems (51, 52, 56, 57, 78). Gene segments that are not included in the table did not have any mutations in their consensus sequence, and share 100% amino acid identity across gene segments.

Farm 2 had more than 99.3% amino acid identity across all gene segments, indicating very few mutations in the quasispecies and no reassortant events and can be seen in Table 6. The PB1 and HA had four mutations. The PB2 and NA had three, and the MP and NS had two. The NP had only one mutation.

**Table 6:** Farm 2 Non-synonymous amino acid mutations.

Segment	Mutations Present	Pigs with mutation	% Identity across Segment
1 – PB2	M64T	2, 17, 18	99.6%
	R299K	5	
	S559N	8	
2 – PB1	M212I	3, 5, 6, 9, 11	99.5%
	I322V	3, 5, 6, 9, 11, 13	
	L708P	5	
	L739P	3, 5, 6, 9, 11	
3 – PA	D396N	8	99.7%
	H437Y	4	
4 – HA	N129T - *Sa	3	99.3%
	S145R - *Ca2	3	
	Q192R - *Sb	21	
	T193A - *Sb	7	
5 – NP	313 V**	ALL	99.8%
	S344L	27	
6 – NA	I19V	3, 5, 6, 9, 11	99.4%
	I128V	7	
	D276Y*	3, 5, 6, 9, 11	
7 – MP	C258S	8	99.4%
	F288V	4, 27	
8 – NS	R88H	3, 5, 6, 9, 11	99.3%
	G183R	17, 18	

The mutations marked *Indicate they are a residue in an antigenic site with the corresponding site abbreviation. **Indicates MxA resistance. Numbering is based on the H3 and N2 numbering systems (51, 52, 57, 78). Gene segments that are not included in the table did not have any mutations in their consensus sequence, and share 100% amino acid identity across gene segments.

A closer look at the antigenic site mutations for HA and NA showed several HA mutations of importance (51, 52). Farm 2 had one Sa mutation: HA-N129T; two Sb: HA-Q192R, HA-T192A; and one Ca_2_: HA-S145R. Fewer NA mutations in antigenic sites were seen, with only one on Farm 2: NA-D276Y (53–55).

Additionally, on Farm 1, three amino acid residues that are associated with increased virulence and confer MxA resistance were present in all pigs: NP-R98K, NP-100I, NP-313 V (except pigs 24 and 30 for R98K) (56, 57). All pigs on Farm 1, except pigs 24 and 30, also had the NP-E53D mutation, which is more than virulent NP-D53E. Farm 2 also had the NP-313 V MxA resistance amino acid in all sites. All amino acid mutations can be found in Tables 5, 6. All nucleotide variations can be found in Supplementary Table S6.

#### 3.3.1 Sequence identity of circulating IAV and vaccines used in sows

**Figure 3:**
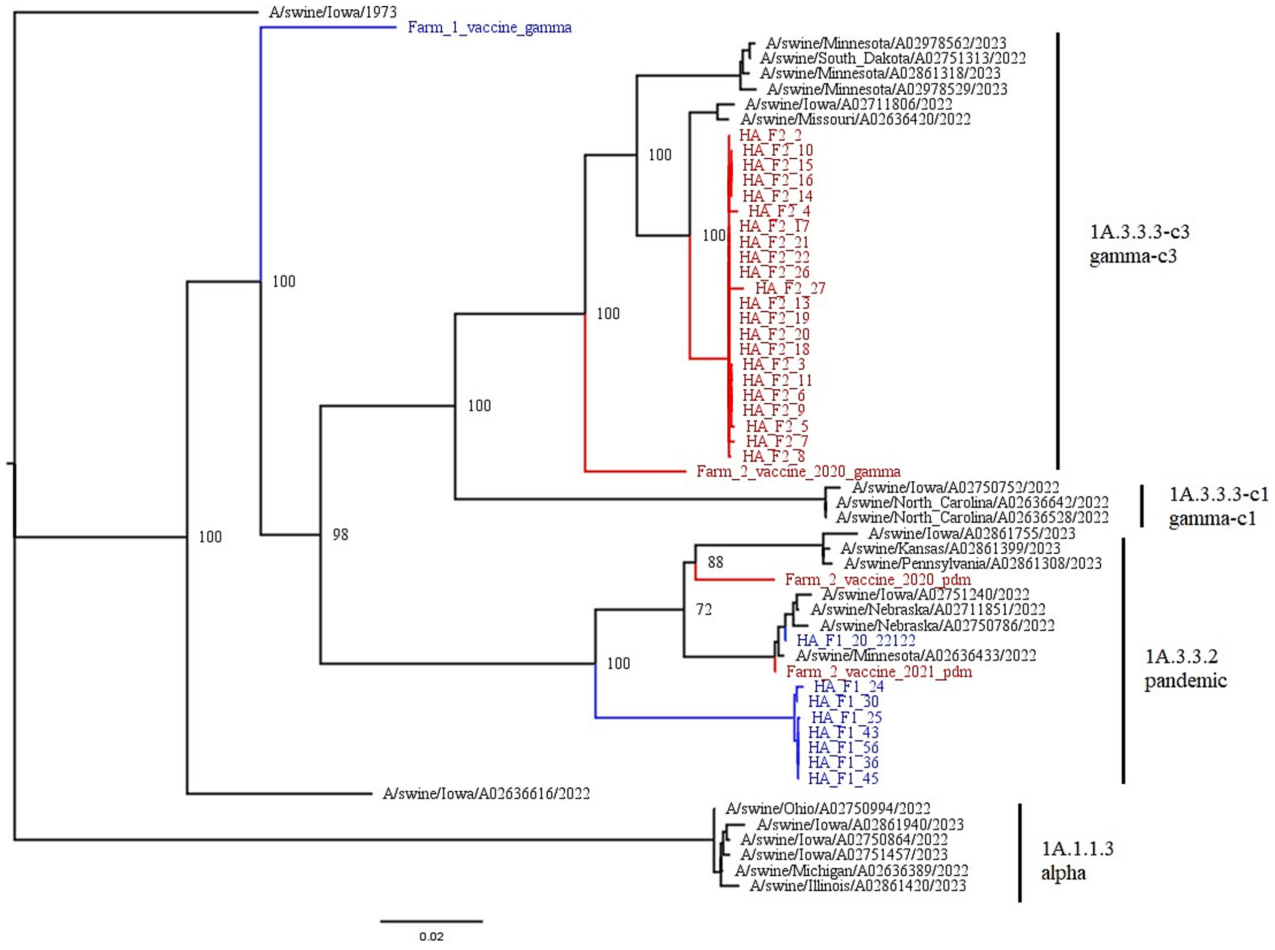
This figure is a phylogenetic tree of the HA gene segments displaying both farms, as well as reference strains selected by the PARNAS algorithm for the phylogenetic analysis. Farm 1 segments are in blue, and falls within the 1A.3.3.2 pandemic clade, and Farm 2 segments are in red and fall within the 1A.3.3.3-c3 gamma-c3 clade. The branches of the tree have been labeled with the major clades with both the global and US nomenclature for HA genes. Vaccines used on both farms are also displayed. F1’s vaccine is in blue, while the three autogenous strains in F2’s vaccine are in red.

**Figure 4:**
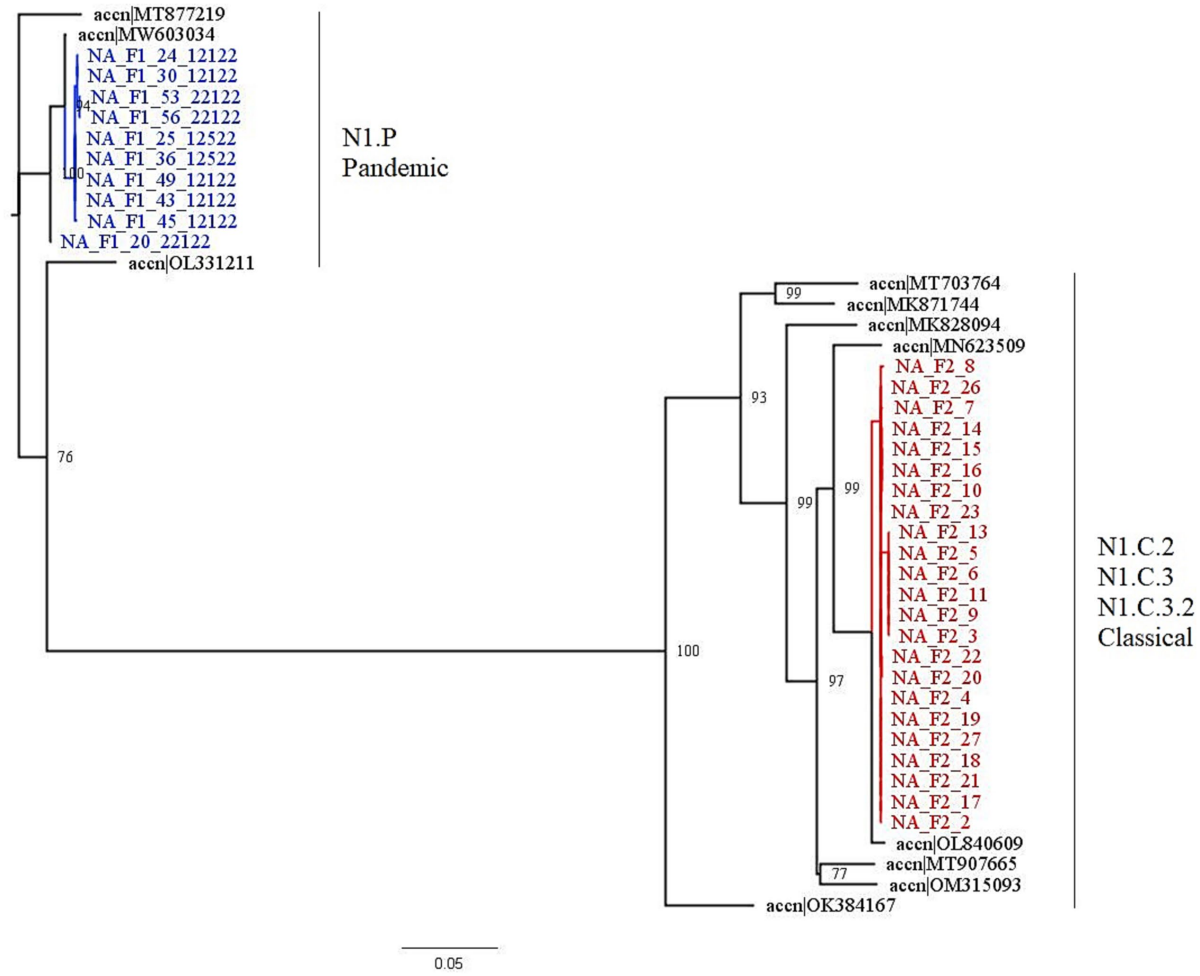
This figure is a phylogenetic tree of the NA1 gene segments displaying both farms, as well as sequences selected by the PARNAS algorithm to represent diverse sequences. Farm 1 segments are in blue, and falls within the N1.P pandemic clade, and Farm 2 segments are in red and fall within the N1.C.3.2 classical clade. The clades for each farm were determined by where the segments were found in the tree. The branches of the tree have been labeled with the major clades with both the global and United States nomenclature for NA genes.

Farm 1 fell into the 1A.3.3.2 pandemic clade, while Farm 2 fell into the 1A.3.3.3c-3 gamma-c3 clade. Figure 3 shows the representative HA clades in current circulation in the United States, with Farm 1 and Farm 2 highlighted in color. The NA1 tree (Figure 4) shows that Farm 1 is a pandemic N1, while Farm 2 is the N1.C.3.2 classical clade. The other six gene segments were analyzed, and their trees can be found in the Supplementary materials. Farm 1’s internal gene constellation is contains all pandemic (P) internal genes (PPPPPP) and Farm 2’s internal genes contain both TRIG (T) and pandemic genes (TTTPPT).

A nucleotide BLAST search of the HA gene shows that of the top 100 hits, and excluding the other Farm 1 viruses, shows mostly (97.8%) human viruses (58). This shows that this is a rare virus to be isolated from swine, and may suggest a recent reverse zoonosis event from humans into the swine population. Additionally, human origin viruses made up the vast majority of top BLAST hits for all gene segments. However, the circulating virus on Farm 1 was most closely related to A/swine/Indiana/A02525081/2021(H1N1) for all segments.

The top nucleotide BLAST search results for Farm 2 show that 100% of the top returns for all gene segments are of swine origin. The hits were also isolated from swine more recently, with most of the strains isolated after 2020. This indicates no recent reverse zoonosis events, and disseminated swine circulation of this virus. Top BLAST hits for both Farms can be found in the Supplementary material – BLAST results.

We compared the HA consensus sequences from each farm to the sequences associated with the vaccines administered to the sows during gestation. Farm 1 used FluSureXP, and the gamma vaccine virus amino acid sequence was 91.9% similar to the circulating 1A.3.3.2 virus. Farm 2 used an autogenous vaccine in their sows, and the 1A.3.3.3-c3 vaccine virus amino acid sequence was 95.9% similar to the circulating 1A.3.3.3-c3 virus.

### 3.4 Statistical results

The results of the mixed effects logistic regression models are presented in Table 7.

**Table 7:** Results of the mixed-effects logistic regression models on the reinfection and shedding of Influenza A virus on swine farms.

Models	Variables	Odds ratio	Estimate	Std. Error	P-value	95% CI low	95% CI high
Model 1 (reinfection)	Wk 1 (Ref.)	–	–	–	–	–	–
	Wk 2	0.154	−1.874	2.947	0.525	−7.649	3.901
	Wk 3	60.846	4.106	1.978	0.038	0.229	7.983
	Wk 4	0.753	−0.286	4.069	0.944	−8.260	7.689
	Wk 5	156.663	5.052	2.684	0.060	−0.210	10.313
	Wk 6	244.612	5.497	2.364	0.020	0.864	10.130
	Wk 7	370.247	5.911	2.484	0.017	1.042	10.780
	Shedding	1377.234	7.225	3.097	0.020	1.155	13.295
	Intercept	0.001	−11.534	4.724	0.015	−20.792	−2.276
Model 2 (shedding)	Wk 1 (Ref.)	–	–	–	–	–	–
	Wk 2	0.071	−2.651	1.166	0.023	−4.936	−0.366
	Wk 3	0.006	−5.094	2.208	0.021	−9.421	−0.767
	Wk 4	0.002	−6.259	2.758	0.023	−11.664	−0.853
	Wk 5	0.002	−6.259	2.754	0.023	−11.657	−0.861
	Wk 6	0.009	−4.695	2.017	0.020	−8.648	−0.741
	Wk 7	0.014	−4.272	1.827	0.019	−7.852	−0.692
	Intercept	8.128	2.095	0.911	0.021	0.310	3.880

Based on the results of the first (reinfection) model, compared to the first week (reference category) the probability of reinfection of pigs with IAV-S was highest in weeks 5, 6, and 7, and reinfection was, as expected, significantly impacted by the shedding of the virus.

The model 2 results (shedding) showed that the probability of IAV-S shedding in weeks 2 to 7 compared to the first week (reference category) was lower.

**Figure 5:**
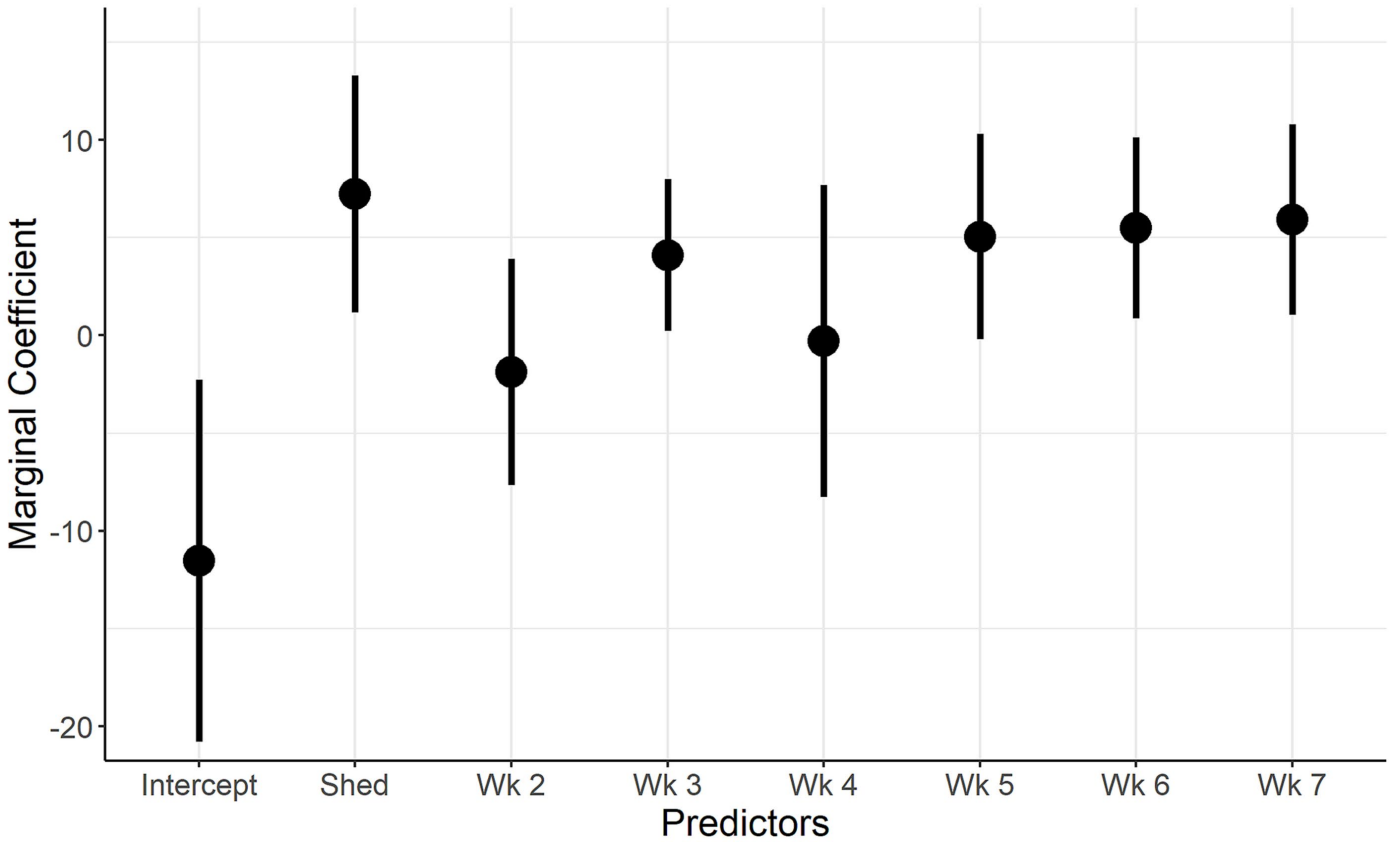
Marginal Coefficient plot for when IAV reinfection is the outcome. Description: The predictors used in the final univariate model (week of sampling, shedding) are on the x-axis and the marginal coefficient values are on the y-axis. The vertical lines represent the 95% confidence intervals of the marginal coefficients.

**Figure 6:**
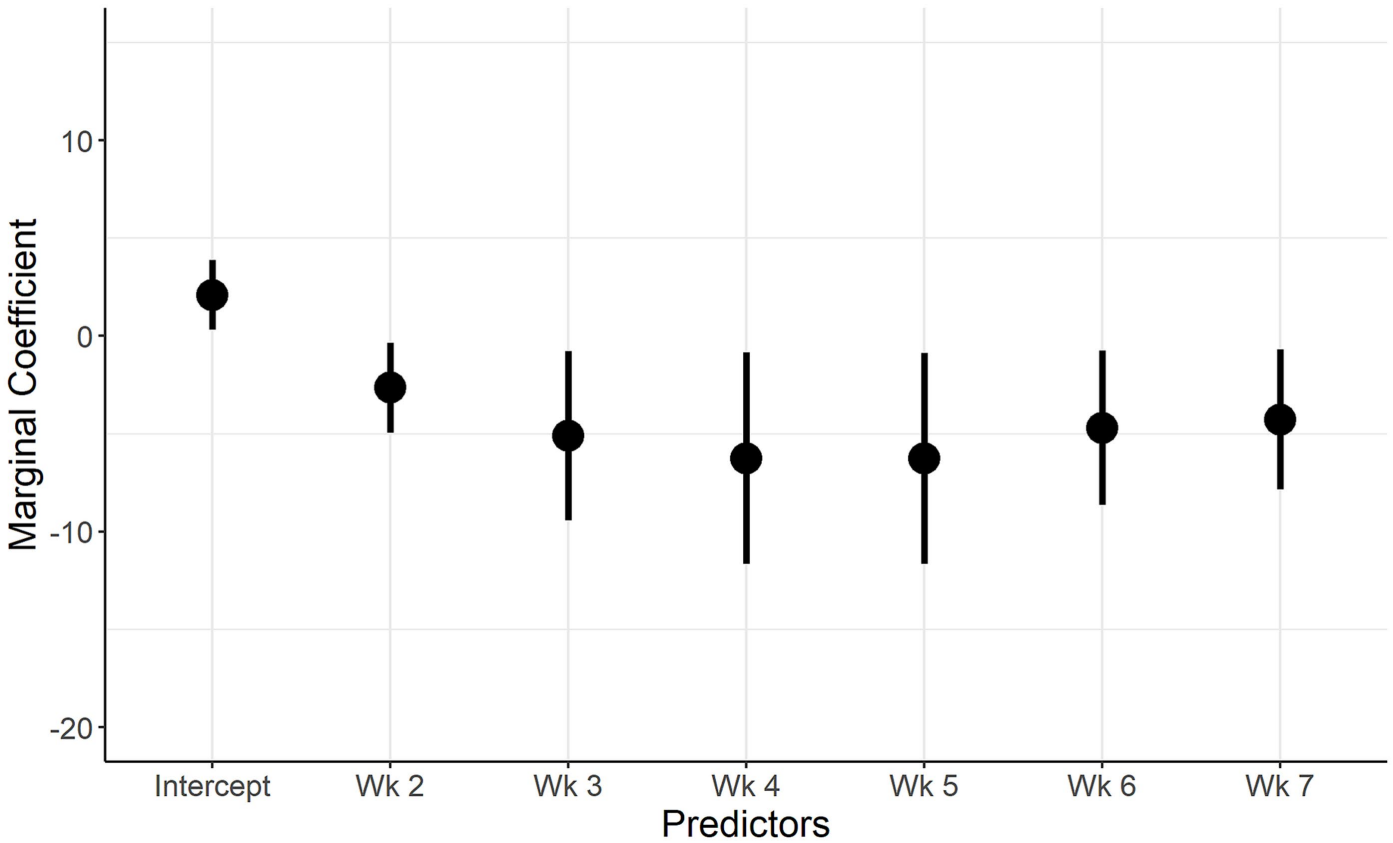
Marginal Coefficient plot when IAV shedding is the outcome. Description: The predictors used in the final multivariate model (week of sampling, farm) are on the x axis, and the marginal coefficient valuesare on the y axis. The vertical lines represent the 95% confidence intervals of the marginal coefficients.

The marginal coefficients and their standard errors for the predictor variables of model 1 and model 2 are presented in Figure 5; Supplementary Figure S1; Figure 6 and Supplementary Figure S2, respectively. In both models, there is evidence of normally distributed residuals based on the QQplot and homogeneity of residuals in both the vertical and horizontal directions.

## 4 Discussion

### 4.1 Key results

Our study reports the pattern of IAV-S shedding by piglets that were naturally infected and reared under commercial nursery conditions. We found that the immediate post-weaning two-week period is an significant time for IAV-S shedding, with 91.2% of piglets with positive nasal swabs in week 1. RT-PCR revealed that only two out of 91 pigs did not shed the virus during the study period. The second model showed that the first week (week of weaning) was the most important week for IAV-S shedding. However, our shedding period was earlier than that of Ferreira et al., who found that shedding was highest around 30 days post placement in the nursery (59). However, our results should be interpreted with the sow vaccination schemes in mind, which were boosted either at week 5 of gestation (Farm 1), or during mid-lactation (Farm 2).

The most likely way of IAV-S spread is nose-to-nose contact or by droplet transmission between infected piglets and susceptible piglets. It should be noted that previous studies established the IAV shedding period in pigs, after contact with infected animals, is highest at 3 days post contact and can last 7 days or more, suggesting these pigs were infected in the immediate peri-weaning period (60, 61).

Co-mingling with other litters, combined with the stress of weaning, feed change, transport and exposure to new pathogens, are all likely contributors to 91.2% of the pigs in our trial to shedding at week 1. Weaning has been shown to increase the incidence of disease in prior studies (62–64). Respiratory pathogens in the post-weaning period, which together compose the Porcine Respiratory Disease Complex, cause damage to nasal mucosa, airways, and interstitium, exacerbating the likelihood for pathology (65). Respiratory illness is often accompanied with post-weaning diarrhea, demonstrating the challenges to piglets’ mucosal surfaces during this transition period (63). Tang et al. reviewed post weaning stress and intestinal health nicely and lend information to the potential pro-inflammatory state pigs of this age (66).

The pigs euthanized during the study may have died as a result of influenza infections, but we cannot confirm or deny if that was the case. We do know that many of the surviving pigs excluded from the final data analysis were shedding during the study period, but due to incomplete sampling over 7 weeks, we were unable to use their results in our statistical analysis. We also assume that the pen mates of the pigs being studied contributed to the influenza circulation, and we are assuming in a similar pattern of shedding to what we saw in our sampled population.

Our study used the gold-standard of individual nasal swabs to describe the shedding patterns of IAV-S, however, this is not a practical method for commercial settings. More commonly, oral fluid collections are used for IAV-S surveillance (67). Many studies have shown the efficacy of oral fluid sampling, however, oral fluid sampling would potentially not be as sensitive or consistent in the later nursery period, as only one of the two farms’ prevalence rose above 9% in the subsequent weeks, which is the threshold for reliable detection in the sample type (68).

One key insight from this study is the variability of timing for IAV-S sampling across herds. As mentioned previously, piglets have been shown to shed later in the nursery period than what was observed in this study. However, other studies have also shown early nursery shedding, and increased disease severity post-weaning (69–71). Understanding sow vaccination protocols, concurrent infections, environmental conditions, weaning age, and other factors will help guide when surveillance efforts should be exercised.

This study reinforces the claim that reinfection events occur post-weaning with the same circulating virus. Reinfection events have been previously reported in several other studies in field conditions (59, 72–74). We found that reinfection events will likely occur in weeks 6 and 7 post-weaning. On Farm 1, 30% (12/40) of piglets became reinfected, while on Farm 2, only 7.8% (4/51) became reinfected. This pattern was seen by Ferreira et al., whose study showed piglets reinfected at 43.2 and 10.7% during the nursery period on different farms (59). Our WGS analysis suggested that only one influenza virus was circulating on each farm during the sampling period, indicating repeated infection with a nearly identical virus. We saw repeated IAV-S detections, with one animal shedding for three consecutive weeks, and four animals shedding three times as both an initial infection and reinfection events, which was also seen in a prior study (75). While our study cannot determine persistent infection versus reinfection, the natural history of influenza virus leads us to believe these secondary shedding events are due to the same pig becoming infected again during the short post-weaning period of our study.

Amino acid analysis identified several mutations that related to antigenic sites on the HA and NA, however, we did not find evidence of fixation in the population. Mutations at antigenic sites are expected, and may be explained by the increased immune pressure at antigenic sites (76, 77). Most viruses from pigs on Farm 1 contained the virulence-boosting NP E53D mutation, which confers MxA resistance. Only two pigs had the opposite NP D53E present. Perhaps this is to be expected, as the E53D mutation suggests higher fitness. Although, those two samples contained the R98K mutation, which has also been shown to play a part in MxA resistance. Additionally, all sequences on Farm 1 had NP-100I and NP-313 V, which are characteristic of pdmH1N1 viruses and expected in pandemic lineage viruses (56). Farm 2 also had the 313 V, presumably because it has a pandemic NP gene, although it has lost the 100I. In all, the mutations found on both farms were not surprising or concerning, considering the quasispecies nature of IAV-S.

Farm 1’s circulating virus was unusual as it contained only pandemic genes. Epidemiologically, these viruses make up a relatively low percentage of circulating strains, as determined by sequencing and analysis performed by the NVSL. Of the 3,668 entries in the OctoFluShow database with WGS information at the time of this publication, only 113 are 1A3.3.2/N1.P/PPPPPP (3.1%) (47). Additionally, nucleotide BLAST search found a nearly identical virus isolated from swine in Indiana, United States in 2021. This highly related virus (top BLAST hit for all gene segments) indicates a possible source or downstream result of infection at the sow farm. However, how that virus was transmitted to Farm 1 is unknown. Farm 1 is a closed herd, and this virus is highly human adapted, so transmissions from humans to the pigs cannot be ruled out. Interestingly, nearly all top BLAST hits were isolated and sequenced from humans in 2013–2016, with no related sequences seen again until the HA gene was sequenced from swine in Indiana in 2020, which provokes questions about where and in what host the virus was circulating between 2016 and 2020. The top 100 BLAST hits can be found in the Supplementary material – BLAST results.

In contrast Farm 2’s H1.gamma-c3/ N1.C.3.2 classic/ TTTPPT is more straightforward in the sense that all of the top 100 hits in BLAST are isolated from swine for all gene segments. Additionally, this virus, or closely related BLAST hits were isolated recently, showing its prevalence in swine populations. This virus appears to be efficient in infecting and transmitting in swine. The virus has swapped some of the internal gene constellations, and this combination of HA, NA, and internal genes represents 7.2% of the viruses with WGS OctoFluShow database.

Farm 1’s commercial vaccine and circulating virus shared 91.9% nucleotide sequence identity. The Figure 3 phylogenetic tree shows that the vaccine is not very closely related to the farm’s circulating strain. The vaccine falls into the H1 gamma clade and would be expected to provide some cross protection between pandemic and gamma isolates. The observed shedding suggests that the antibody levels present either did not provide cross protection against shedding, or the antibody levels were too low to provide protection against shedding. However, antibody analyses from the Farms will be addressed in a separate publication, and further recommendations surrounding vaccinations will be made there. In regards to a higher reinfection rate, we can only speculate the cause in the absence of antibody titer information, and could be attributed to decay of maternal antibodies, insufficient piglet immunity following first exposure (if maternal antibodies were at low levels), or other virus specific virulence factors or environmental differences on Farm 1.

In contrast, Farm 2 had three isolates in their autogenous vaccine that fell within the H1 tree. Although two pandemic clade H1 antigens are included, there is also a very closely related gamma-c3 isolate. We speculate that this strain provides protection against IAV-S, but antibody analysis is needed to support this speculation further.

On both Farms, IAV-S was in circulation, and we expect sows to mount an immune response to infection. However, we did not sample sows in this study, therefore sow antibody analysis will not be performed in our subsequent analysis.

In summary, we observed two nurseries naturally infected IAV-S and found that viral shedding was highest in the early post-weaning period, with reinfection events in 30% of animals on one farm. We also sequenced the viruses in circulation on both farms and did not find significant mutations during the sampling period, and only one virus in circulation on each farm. However, we did see that the Farm 1 virus is likely a recent reverse zoonosis, while the Farm 2 was a swine virus.

### 4.2 Limitations

This study population was chosen purposefully to monitor influenza transmission in naturally exposed commercial farms. We expected influenza shedding at all time points in the study. We did not manipulate immunizations (either sow or piglet) from typical farm protocols, antimicrobial treatments, or other control measures on the farm that may have impacted influenza transmission during the study.

We did not assess serum or mucosal antibody levels in this analysis, and that data will be addressed in a separate analysis. Maternal antibody interference with piglet antibody production has been documented for IAV in swine (16, 73, 74). As both sow farms were vaccinated during each gestation and were exposed to circulating virus, maternal antibody interference may play a role in the initial infection and reinfection events seen in Farms 1 and 2, and will be assessed in a separate analysis.

IAV-S infections infect pigs for a duration of illness of 7 days. However, shedding can be shorter or longer than the 7 day sampling interval we chose. We saw several pigs who shed for two to three weeks consecutively, but we may have missed pigs that shed for less than 7 days. Because of the 7 day sampling interval, we may have mislabeled prolonged shedding events that may have been reinfection events.

## 5 Conclusion

In conclusion, we studied IAV-S shedding in two commercial nursery populations. We found that shedding occurs most frequently in the first week post weaning. We support previous findings that reinfection events in the nursery period occur and that the events are attributed to reinfection with the same circulating virus, and was not due to a new virus introduction. We also analysed the mutations present in the circulating viruses on each farm. We found that Farm 1’s virus was a recent reverse zoonosis event, previously isolated once in 2021. We found that shedding events were not different between the two farms sampled, but that reinfection events occurred more frequently on one farm compared to the other.

Our findings suggest that testing of postweaning pigs for IAV-S should include the first 2 weeks post-weaning. Pen-based testing is appropriate, rather than a targeted sampling of clinical pigs, given the high prevalence of piglet shedding during these periods, and the practicality of pen-based sampling. Further studies are needed to correlate the contribution of maternal immunity in commercial settings on piglet IAV-S shedding and viral transmission.

## Data Availability

The datasets presented in this study can be found in online repositories. The names of the repository/repositories and accession number(s) can be found in the article/Supplementary material.
