# Supplementary material for: Influenza A virus shedding and reinfection during the post-weaning period in swine: longitudinal study of two nurseries
Source: Front Vet Sci. 2024 Nov 13;11:1482225. doi: 10.3389/fvets.2024.1482225 (PMC11601151; doi:10.3389/fvets.2024.1482225)

Supplementary Material

Influenza A Virus shedding and reinfection during the post-weaning period in swine: longitudinal study of two nurseries

Suzanna Storms^1,2^, Tara Prezioso^2^, Csaba Varga^2^, Antonio Leonardi-Cattolica^1,2^, Leyi Wang^1^, James Lowe^1*^

*** Correspondence:** Corresponding Author: jlowe@illinois.edu

# Supplementary Data

## RNA Extraction

The Wash 1 plates were prepared with 500 μL of provided wash buffer per well. The Wash 2 and 3 plates were prepared with fresh 80% ethanol, using 500 μL per well in plate 2, and 250 μL in plate 3. The elution plate was prepared with 50 μL of provided elution solution.

The sample plate was prepared last. 5 μL of proteinase K was added to each well, and then the plate was moved into the biosafety cabinet, where 200 μL of vortexed sample was added. Then, premixed binding solution (265 μL), magnetic beads (10 μL), and Xeno RNA extraction control (2 μL) was added to each well, for a total of 277 μL of binding mixture per well. 200 μL of PBS was used in an extraction control well instead of sample. After samples were prepared, all plates were placed onto the Kingfisher Flex instrument and run using the MVP_Flex factory protocol. After the protocol was complete, plates were removed, and the eluate was immediately used for PCR. Per the manufacturer's instructions, the unused eluate was covered and stored at -20°C.

## RT-PCR of nasal swab samples

For each reaction, 12.5 μL of 2x Mastermix buffer, 2.5 μL of PCR enzyme mix, 1 μL of Primer Probe mix, and 1 μL of nuclease-free water were prepared, for a total volume of 17 μL. Prepared mastermix was loaded into a 96 well MicroAMP Optical PCR plate (Applied Biosystems, Waltham, MA, USA) and transported to a sample loading area separate from the PCR preparation area. In the sample loading area, 8 μL of freshly extracted sample was added to each well. Two positive controls were run per plate in addition to a PBS extraction control and a negative control (nuclease-free water). An optical film cover (Applied Biosystems, Waltham, MA, USA) was added to the plate before transport. The plate was then centrifuged for 1 minute at 200 RCF.

All PCR reactions were performed on QuantStudio 3 Real-Time PCR system with QuantStudio Design and Analysis software version 1.5.2 (Applied Biosystems). The FAM channel (SIV RNA) and the VIC channel (for Xeno controls) were selected, along with ROX passive reference dye, which was included in the RT-PCR buffer. The Taq program was chosen, with reverse transcription at 48°C for 10 minutes and RT inactivation/initial denaturation at 95°C for 10 minutes. The amplification step was 40 repetitions of 95°C for 15 seconds, followed by 60°C for 45 seconds.

## Whole Genome Sequencing

After targeted amplification, the cDNA was purified by the QIAquick PCR purification kit (Qiagen, Venlo, Netherlands), and 40uL was eluted into the provided buffer EB. The manufacturer’s instructions were followed. Briefly, 50 μL of PCR product was added to 250 μL of buffer PB. 3-5 μL of glacial acetic acid were added to the reaction tube if the solution was not yellow, as measured by the provided pH indicator. The sample was applied to the provided spin columns and centrifuged at 13,000 rpm for 1 minute, followed by discarding of the flow-through. 750 μL of buffer PE was added to the spin column and centrifuged at 13,000 rpm for 1 minute, flow through was discarded, and the one minute centrifugation was repeated to eliminate any liquid from the spin column. The spin column was moved to a clean microcentrifuge tube, and 42 μL of EB buffer was added to the center of the spin column membrane and incubated for three minutes at room temperature, followed by centrifugation at 13,000 rpm for one minute. The purified sample was stored at -20ºC until used for sequencing.

## Supplementary Figures

**Supplementary Figure 1, A-F.** These figures are phylogenetic trees of the gene segments displaying both farms, as well as sequences selected by the PARNAS algorithm to represent diverse sequences. Farm 1 segments are in blue, and Farm 2 segments are in red. The clade or constellation for each segment was determined by where the segments were found in the tree. The trees have been labeled with the major clades or constellations pertaining to each segment. The figures are (A) PB2, (B) PB1, (C) PA, (D) NP, (E) MP (F) NS.

(A)


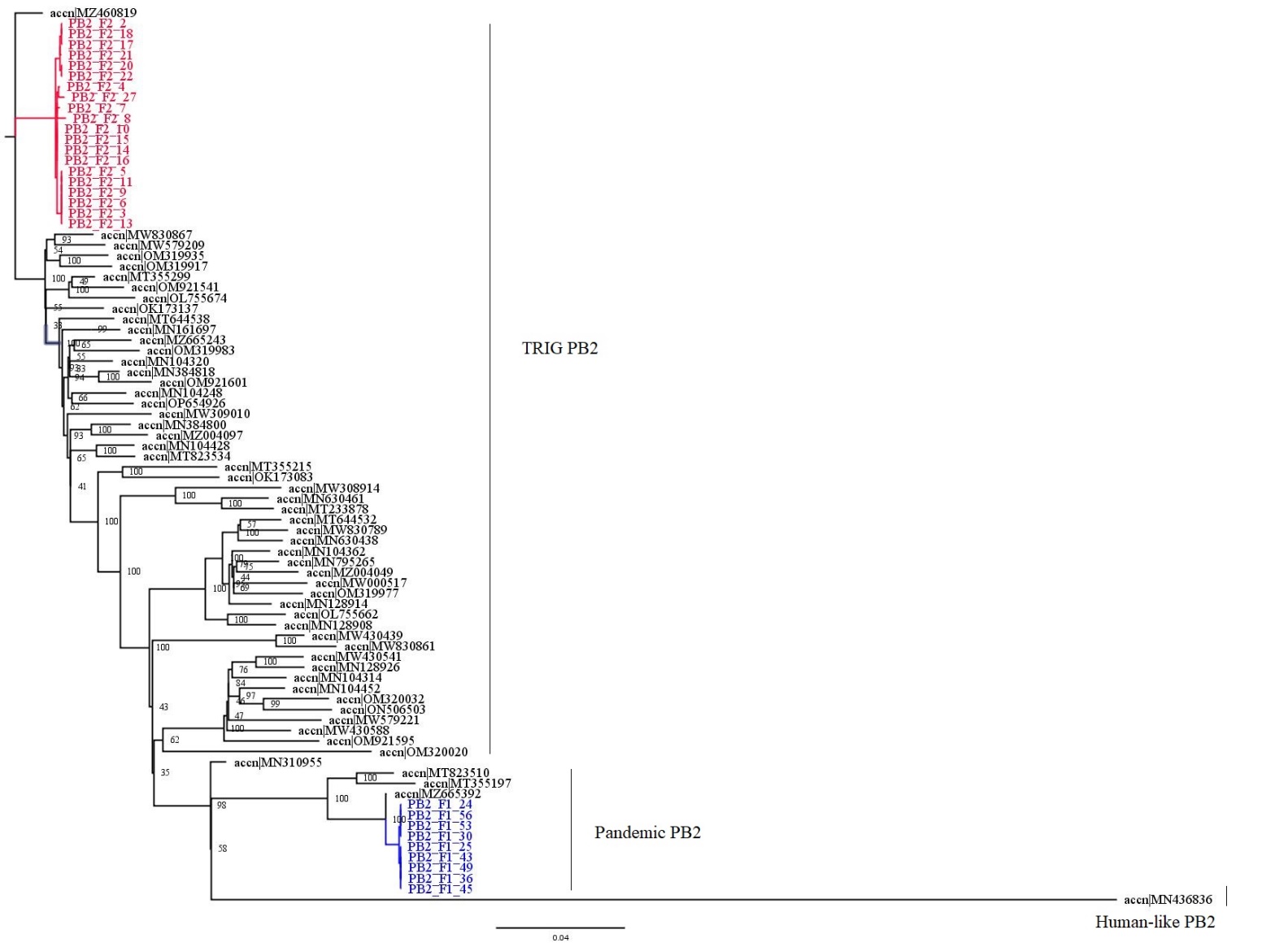


(B)


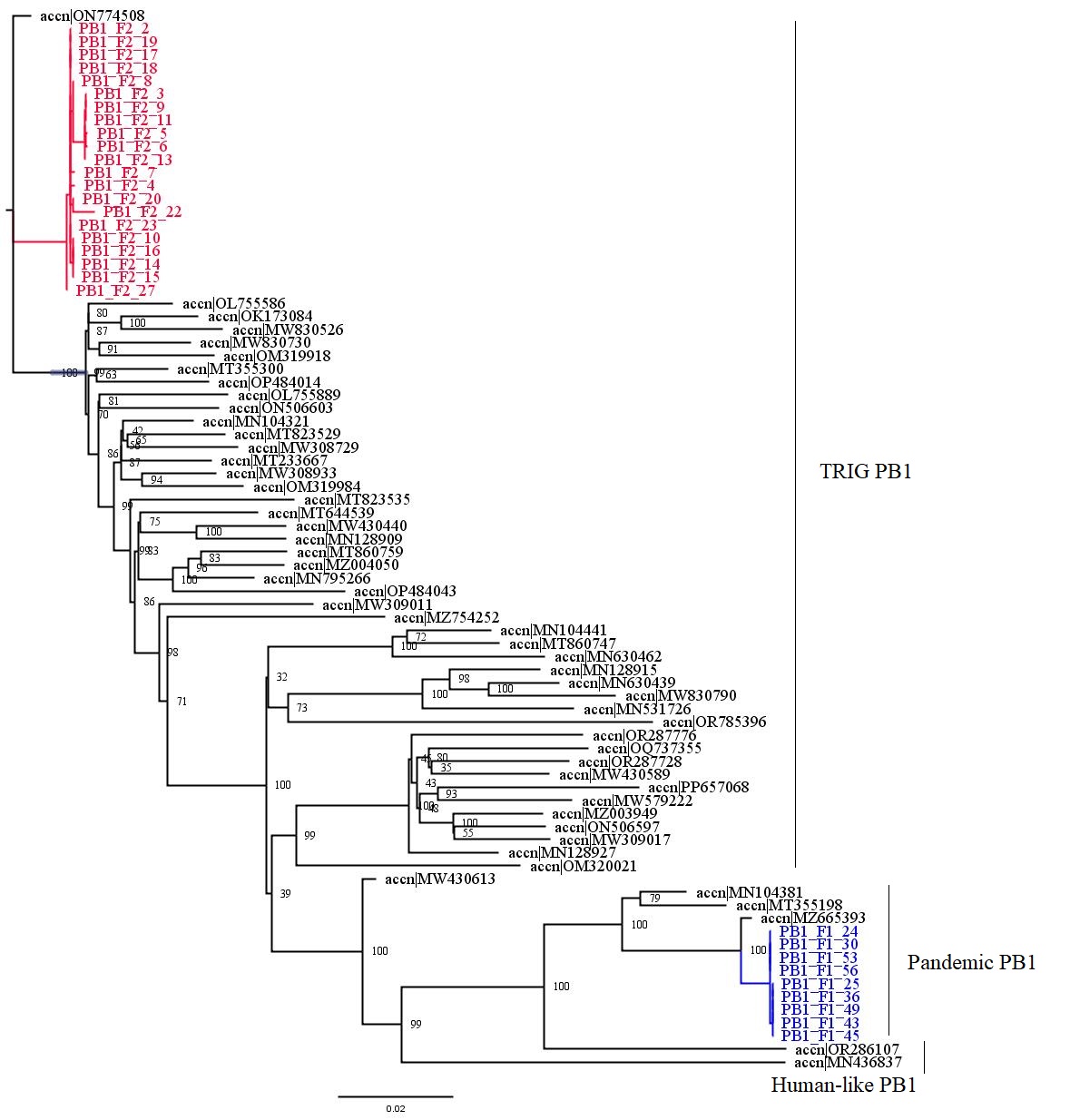


(C)

**
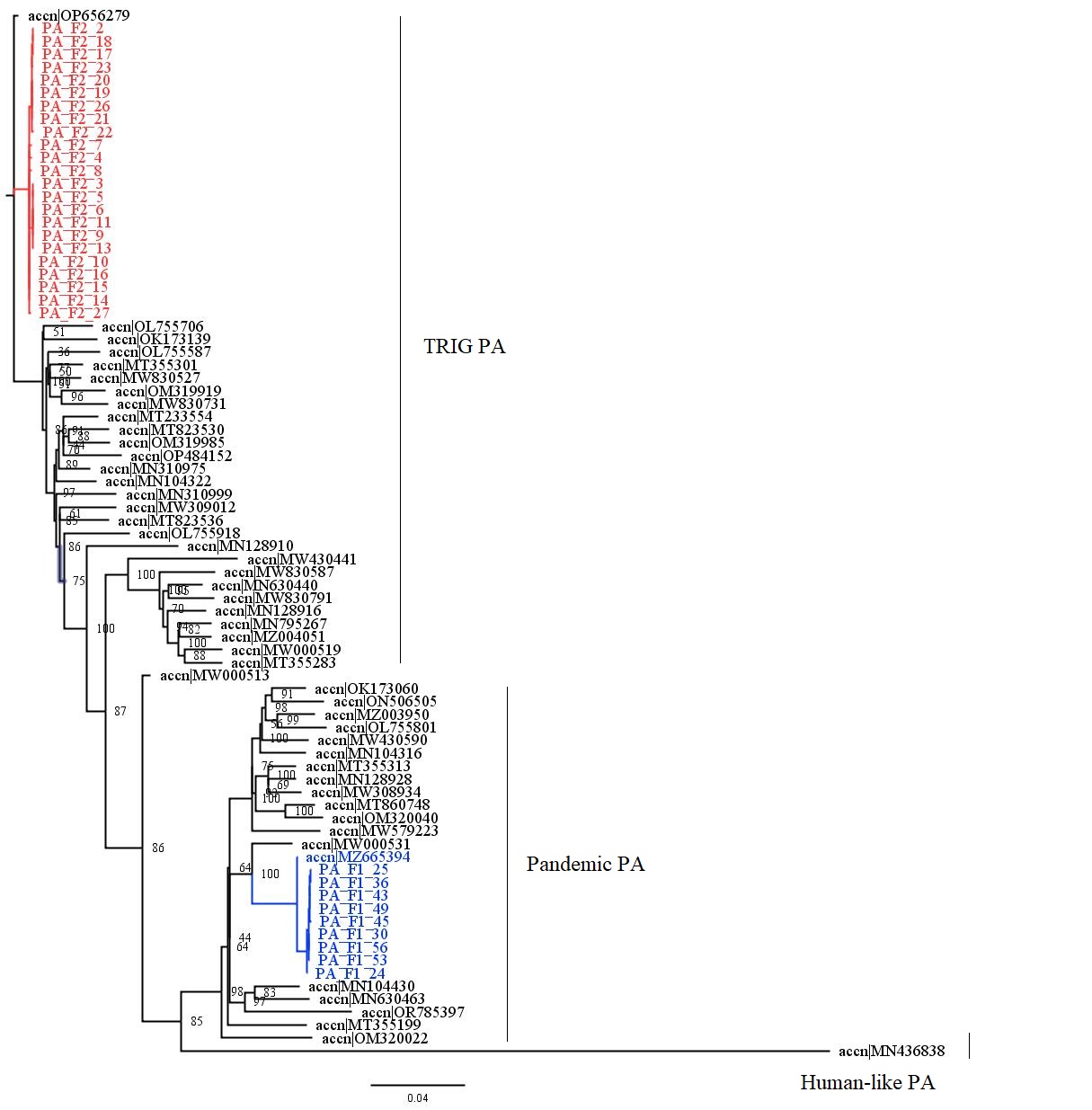
**

(D)


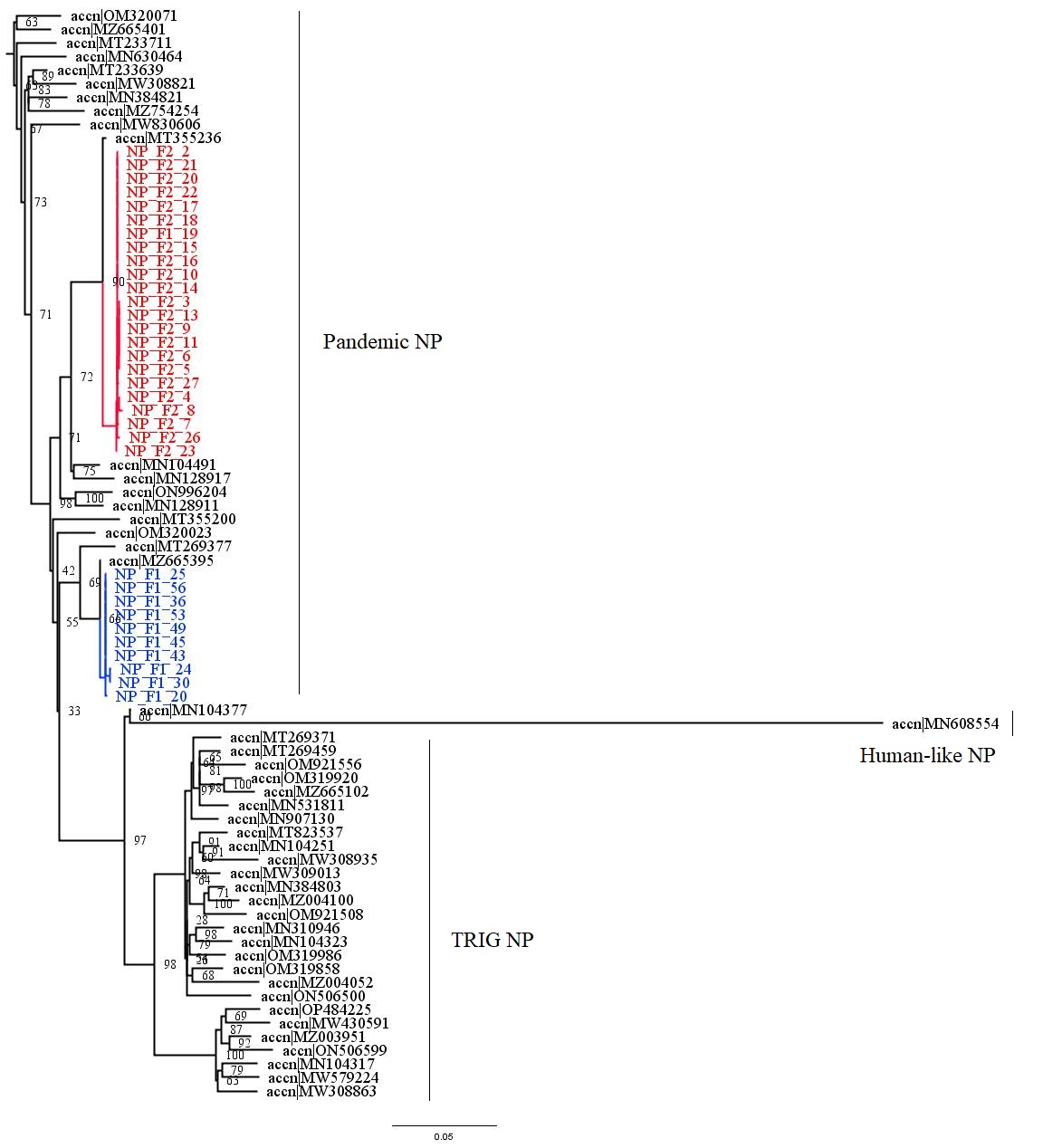


(E)


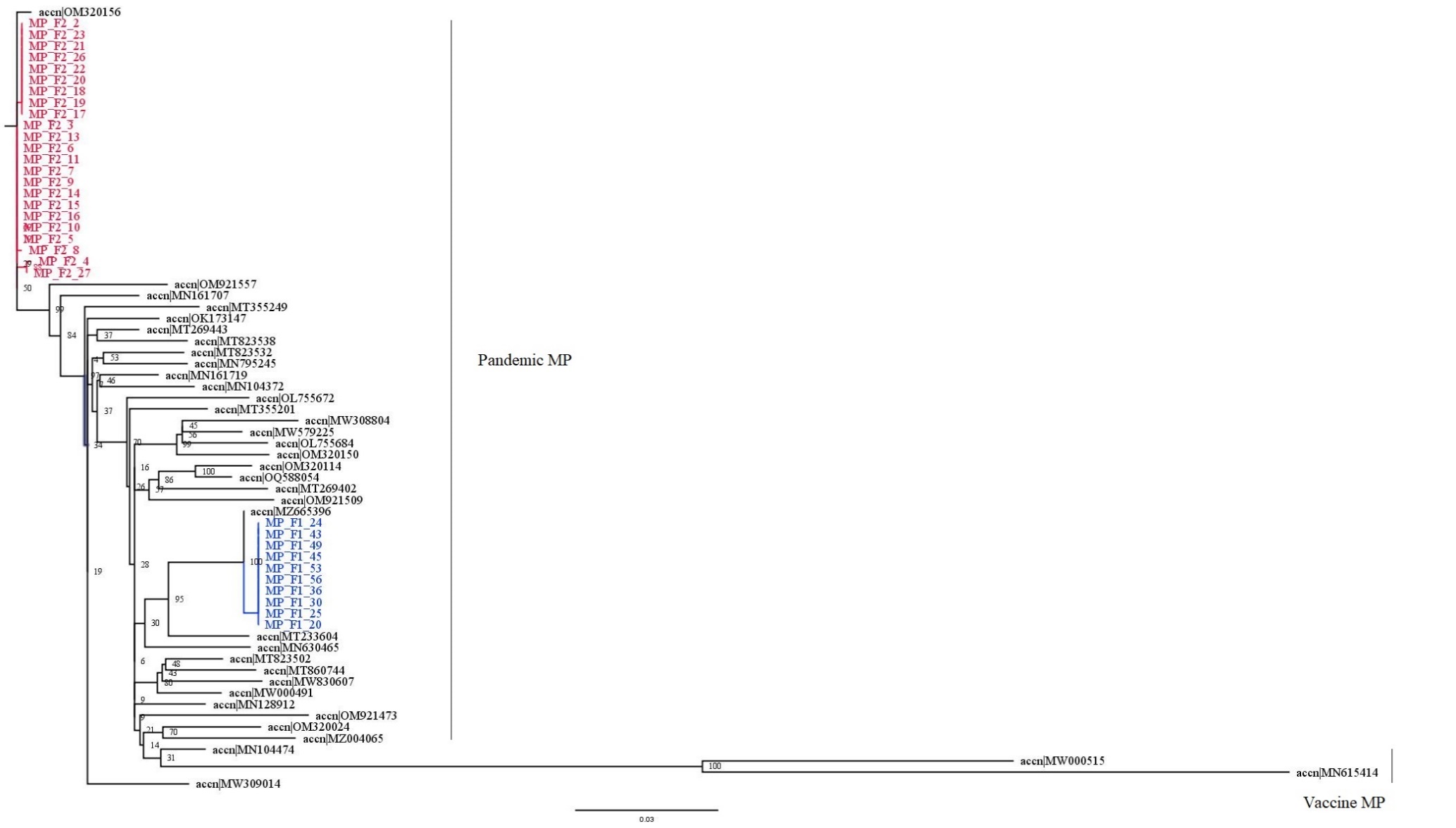


(F)


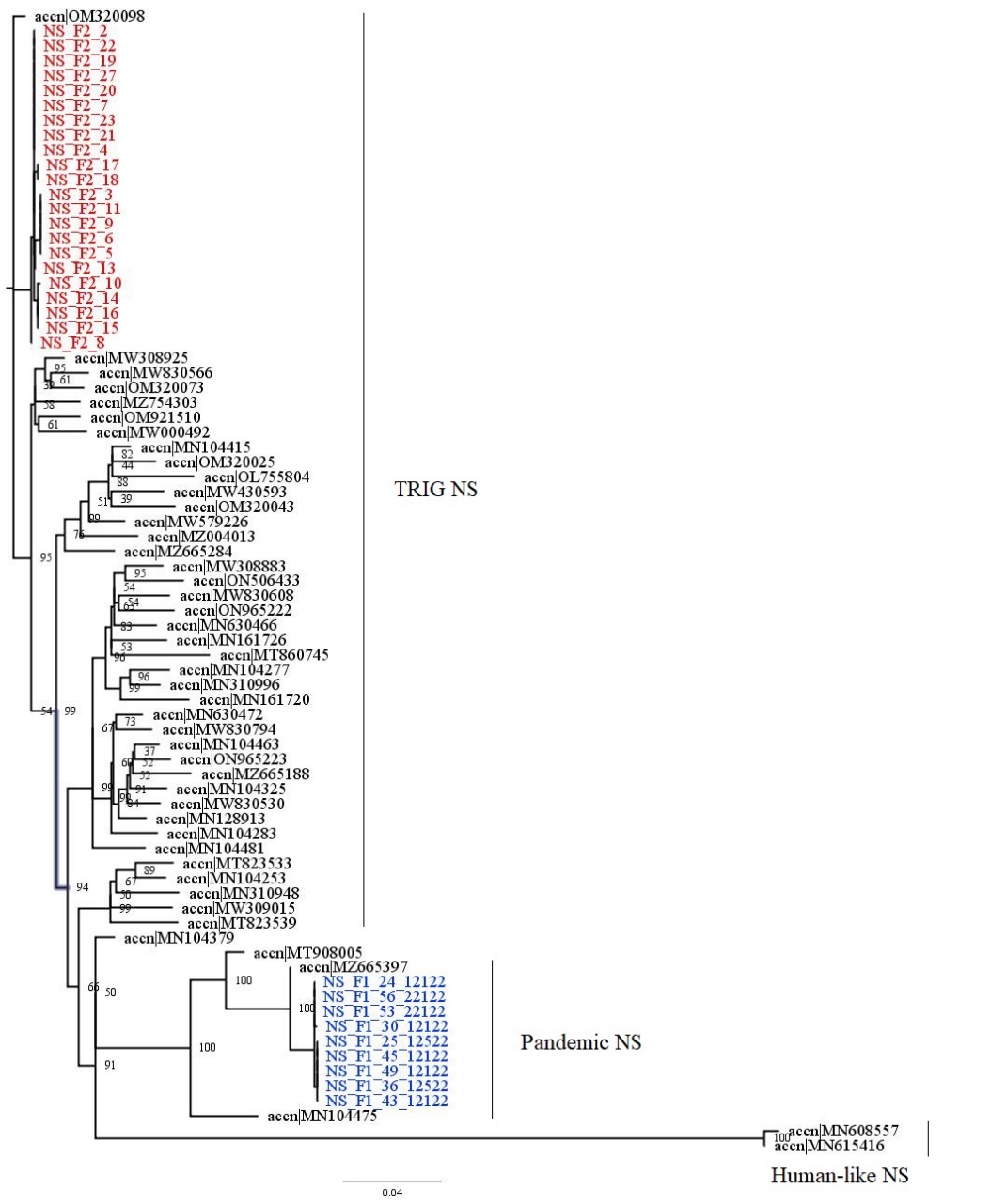

Supplement: Supplementary file 2 [file Data_Sheet_1.docx]
